# Directional Coordination of Thumb and Finger Forces during Precision Pinch

**DOI:** 10.1371/journal.pone.0079400

**Published:** 2013-11-13

**Authors:** Ke Li, Raviraj Nataraj, Tamara L. Marquardt, Zong-Ming Li

**Affiliations:** Hand Research Laboratory, Departments of Biomedical Engineering, Orthopaedic Surgery, and Physical Medicine and Rehabilitation, Cleveland Clinic, Cleveland, Ohio, United States of America; The University of Queensland, Australia

## Abstract

The human opposable thumb enables the hand to perform dexterous manipulation of objects, which requires well-coordinated digit force vectors. This study investigated the directional coordination of force vectors generated by the thumb and index finger during precision pinch. Fourteen right-handed, healthy subjects were instructed to exert pinch force on an externally stabilized apparatus with the pulps of the thumb and index finger. Subjects applied forces to follow a force-ramp profile that linearly increased from 0 to 12 N and then decreased to 0 N, at a rate of ±3 N/s. Directional relationships between the thumb and index finger force vectors were quantified using the coordination angle (CA) between the force vectors. Individual force vectors were further analyzed according to their projection angles (PAs) with respect to the pinch surface planes and the shear angles (SAs) within those planes. Results demonstrated that fingertip force directions were dependent on pinch force magnitude, especially at forces below 2 N. Hysteresis was observed in the force-CA relationship for increasing and decreasing forces and fitted with exponential models. The fitted asymptotic values were 156.0±6.6° and 150.8±9.3° for increasing and decreasing force ramps, respectively. The PA of the thumb force vector deviated further from the direction perpendicular to the pinching surface planes than that of the index finger. The SA showed that the index finger force vector deviated in the ulnar-proximal direction, whereas the thumb switched its force between the ulnar-proximal and radial-proximal directions. The findings shed light on the effects of anatomical composition, biomechanical function, and neuromuscular control in coordinating digit forces during precision pinch, and provided insight into the magnitude-dependent force directional control which potentially affects a range of dexterous manipulations.

## Introduction

The human opposable thumb is a remarkable product of evolution of the hand freeing from walking requirements. Thumb-finger opposition enables the hand to perform fine manipulation of objects with well-coordinated digit forced. The directions of digit force vectors play an important role in dexterous manipulation. The thumb and fingers adjust their force vector directions in accordance with the mechanical properties (e.g. mass, shape, or surface friction) of a grasped object [1]. Different types of manual tasks such as lifting and holding also influence the directions of digit force vectors [2]–[4]. It has been shown that the force directions and the force magnitudes are combined to minimize the net force and the net moment for stable holding of a freely moveable object [5]. Moving an object from one location to another, the force vectors were oriented in the direction of movement to efficiently accelerate the object [6]. These studies serve as examples to demonstrate how the digits control their force vector directions to successfully execute manual tasks.

Compared to lifting and holding tasks, applying force on an externally stabilized object does not require static or dynamic force equilibrium, therefore allowing digits to employ different strategies of force coordination [7]. It has been shown that the digit force vectors during force application tend to deviate from the direction perpendicular to the contact surface [8]. The force vector deviations were exacerbated in chronic stroke patients [9] or elderly individuals [10], suggesting decreased capacity of controlling the force directions in these populations. Therefore, the digit force direction during force production provides valuable information about manual functions. In these studies, however, the force vector deviation was examined when the exerted force magnitude was steadily maintained or when the magnitude was gradually increased within a limited range. It remained unknown whether the force directions were invariant across force levels and whether the force directions differed with respect to force exertion versus force relaxation.

Many manipulative actions entail precise pinch grasp with the pulps of the thumb and the index finger. Despite extensive previous studies on precision pinch, the directional relationship between the force vectors of the thumb and the index finger is not well established. Given that the thumb and the index finger differ with respect to anthropometry (e.g. length, size, shape), anatomy (e.g. bone, joints, tendons and muscles), and neurophysiology (e.g. tactile afferents, nerve innervations and motor cortical representations), it is intriguing to understand how the two digits natural coordinate their force vectors during precision pinch and to what extend the force vectors oppose each other.

The purpose of this study was to investigate the directional coordination of the force vectors applied by the thumb and the index finger during a precision pinch when the pinch forces increased and decreased. In order to examine the intrinsic relationship between the force vector directions and the force magnitudes, pinch forces were applied on an externally secured apparatus so that the task does not require the subjects to inherently abide by static or dynamic equilibrium during grasp. The force vector directions were examined using the coordination angle (CA) between the two digit force vectors, the projection angles (PAs) of each digit force vector with respect to the object's surfaces, and the shear angles (SAs) within the pinch surface of the object. The dependence of these angles on force magnitude was analyzed under both increasing and decreasing force conditions. Based on the anthropometrical, anatomical, and neurophysiological differences between the thumb and the index finger, we expected that each digit would have a digit-specific tendency of force vector application at the initial phase of pinch and they would demonstrate synergistic force vector coordination with increasing pinch forces. Therefore, we hypothesized that the CA between the two force vectors would be dependent on the pinch force magnitudes. Applying increasing pinch forces is associated with object lifting and decreasing pinch forces is associated with object release, we hypothesized that the force vector coordination pattern would be different for force increasing or decreasing. Furthermore, we hypothesized that the relationship between force magnitude and force direction quantified by PAs and SAs are digit-specific in contributing to the CA.

## Materials and Methods

### Ethics Statement

Informed, written consent was obtained from each subject prior to the study. This study was approved by the Institutional Review Board at the Cleveland Clinic Foundation.

### Subjects

Fourteen right-handed, male subjects (Age: 29.1±4.3 y; Height: 174.9±5.7 cm; Weight: 76.2 ±10.8 kg) participated in this study. No subject reported a history of musculoskeletal or neurological disorders in their hands or their upper extremities. All subjects had normal or corrected-to-normal vision.

### Experimental set-up

**Figure 1:**
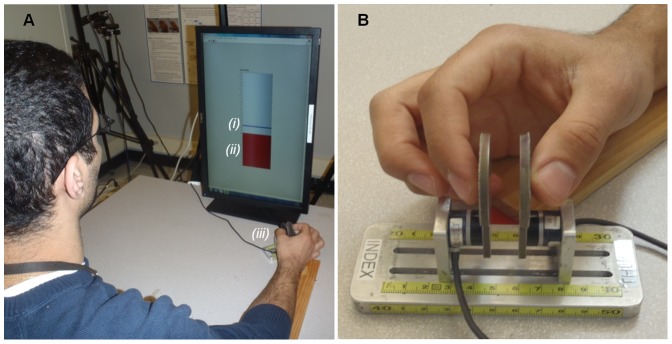
Experimental setup for the pinch task. (A) A subject interfacing with the pinch apparatus while viewing the monitor for force feedback. *(i)* The horizontal line in the middle of the tank serves as the target line; *(ii)* the vertical bar indicates the real-time pinch force of the subject; and *(iii)* the pinch apparatus in the experiment. (B) Close-up of the typical pinching posture assumed while the digits are interfacing with the apparatus.

A pinch apparatus with two parallel pinch surfaces was designed to measure fingertip forces at each digit-object interface using two six-component force/torque transducers (Nano17, ATI Industrial Automation, Inc., Apex, NC) (**Figure 1**). The signals were amplified and multiplexed using a custom ATI interface box (ATI Industrial Automation, Inc., Apex, NC) and digitized by a 16-bit analog-digital converter (PXI-6289, National Instrument, Austin, TX). The twelve force/torque signals from the two transducers were recorded simultaneously at a sampling frequency of 1000 Hz.

The pinch apparatus was rigidly fixed to a testing table at a distance of 35 cm from the table edge. The pinch surface planes were obliquely oriented approximately 45° with respect to the table edge (**Figure 1A**). The pinching surfaces were covered with 100 grit sandpaper and had a grip span of 2 cm. A 22-inch computer monitor, with a viewing area of 1024×1280 pixels, was positioned 50 cm from the edge of the table. Graphically displayed in the center of the screen was a tank indicator with overall dimensions of 300 (width) ×800 (height) pixels. A range from 0 to 15 N was scaled along the vertical length of the tank. The tank contained a pinch force bar for visualization of which the height represented the average real-time normal force exerted onto the pinch apparatus by the thumb and the index finger of the subject. A horizontal target line (5.3 pixels in width), programmed to move vertically, was also displayed within the tank to cue the subject as to how much pinch force to generate at a given instant during each trial(**Figure 1A**). The interface and the data collection were implemented using a custom *LabVIEW* program (National Instrument, Austin, TX).

### Test procedures

Each subject sat comfortably in a height adjustable chair at the testing table with the pinch apparatus aligned with their right shoulder. The right upper arm was approximately abducted 30°in the frontal plane and flexed 20°in the sagittal plane. The elbow was at about 60° of flexion. The forearm was placed on a support and in a neutral pronation/supination position. Subjects were then instructed to pinch the apparatus with the pulps of their thumb and index finger to follow the target line displayed on the tank indicator (**Figure 1B**). For each trial, the target line linearly rose (ramped up) from 0 to 12 N and then immediately began to descend (ramped down) to 0 N at a force rate of 3 N/s. The entire up/down ramp profile occurred over an 8-s pinching duration. Subjects were instructed to apply pinch force onto the apparatus to control the height of the pinch force bar such that it matched as closely as possible to the moving target line. At the commencement of each trial, when the target line was at 0N, the subject was instructed to position the thumb and the index finger proximate to each respective pinch plate without making contact with the plate. Each subject performed 10 trials with 1 minute rest intervals between trials to minimize the effects of muscle fatigue.

### Data analysis

**Figure 2:**
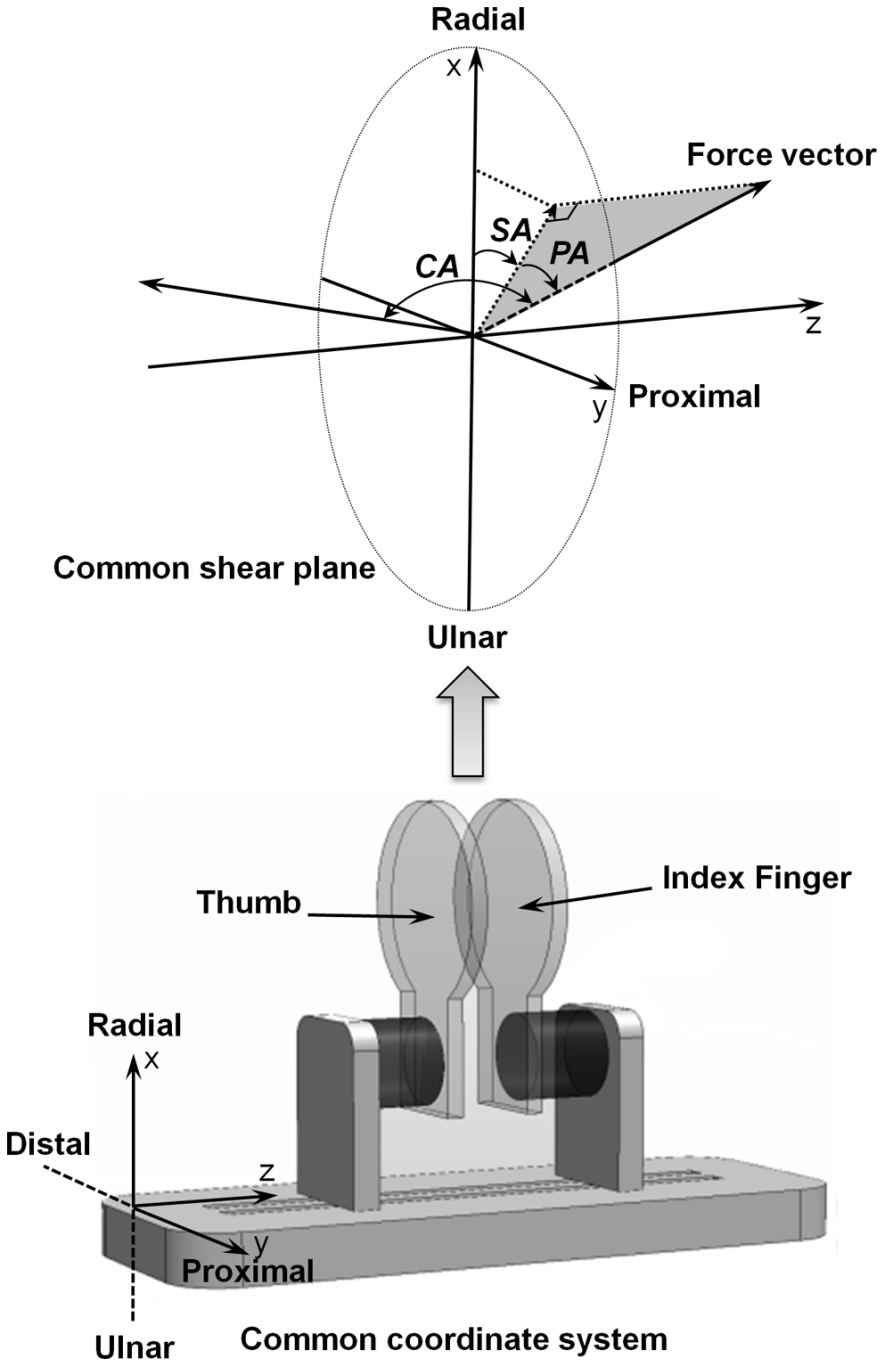
Definition of coordinate system and angular parameters.

From trial to trial, the pinch force profiles showed variant turning points from the ramp-up phase to the ramp-down phase. To ensure that the data examined for each trial had the same force magnitude, analyses were performed on the data for pinch forces between 0 and 10 N**.** The three forces recorded from each transducer were low-pass filtered with a cut-off frequency of 20 Hz. Then, the force components were transformed to a common coordinate system presumed to be affixed to the base of the pinch apparatus. A common shear plane was defined by the common coordinate system as the *x*-*y* plane, such that positive *x* directed radially, and positive *y* directed proximally (**Figure 2**). The procedures of the coordinate transformation included three steps: first, three non-collinear points on the apparatus base were digitized using a MicroScribe device (MicroScribe G2X, Immersion Corporation, San Jose, CA); second, the relationship of each transducer coordinate system to the digitizer coordinate system was established using simultaneous digitization and center of pressure (COP) measurement; third, the transformation matrix from each local transducer coordinate system to the common coordinate system were determined according to standard matrix transformations [8].

The force directions of the thumb and the index finger during increasing and decreasing pinch force ramps were assessed using the following metrics: 1) the coordination angle (CA) between the force vectors of the two digits; 2) the projection angle (PA) of each force vector with respect to the x-y plane; 3) the shear angle (SA) of each vector within the x-y plane (**Figure 2**). The CA was defined as the angle formed by the thumb and the index finger vectors and calculated as follows:
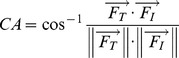
(1)


where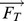 and
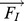
are the 3-D force vectors of the thumb and the index finger, respectively, expressed in the common coordinate system.

The dependence of the CA on the pinch force (PF) was described using the following first-order exponential regression function:
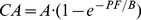
(2)


where A is the upper limit of CA, and B is the ‘force constant’ parameter indicating the rate at which CA moves towards its upper limit. Specifically, B represents the force at which the CA reaches 63% of its upper limit. This function was chosen for regression fitting to include CA versus PF profile features that were observed during pilot testing sessions. These features included CA approximately equal to zero at PF equal to zero, apparent first-order monotonic rise of mean CA with increasing PF, and rise towards clear and distinct upper limit value, effectively serving as an asymptote.

The PA of each force vector was defined as the angle of the force vector with respect to the *x*-*y* (shear) plane (**Figure 2**), i.e.
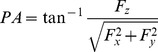
(3)


The SA quantified the angular orientation of each vector's projection with respect to the x-axis of the shear plane (**Figure 2**), and was defined as
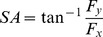
(4)


The intensity distribution of the shear force applied by the thumb and the index finger throughout the shear plane was demonstrated by a tuning curve [11]. The shear plane was equally divided into 16 directions, 22.5° for each segment. For the increasing and decreasing force conditions, the average shear force magnitudes within each segment were calculated as representative values for that direction. A closed-form curve was used to connect the points for each condition trace and for each digit to illustrate the distribution of the shear force.

### Statistical analyses

Statistical analyses were performed using SPSS (SPSS Inc., Chicago, IL). Data were assessed for normality using the Kolmogorove-Smirnov test. The pinch forces, which ranged from 0-10 N, were divided equally into 10 levels. Two-way repeated measures ANOVA with a Greenhouse-Geisser correction was performed to determine the effects of force levels and force conditions (i.e., increasing and decreasing force) on the CA. For the outcome measures of PA and SA, three-way repeated measures ANOVA with a Greenhouse-Geisser correction were performed to evaluate the effects of digits, force levels, and force conditions. Post hoc tests with the least significant difference were used for all pairwise comparisons. Paired *t*-tests were performed to evaluate the effects of increasing versus decreasing pinch force on the regression parameters. A *p*-value of less than 0.05 was considered statistically significant.

## Results

**Figure 3:**
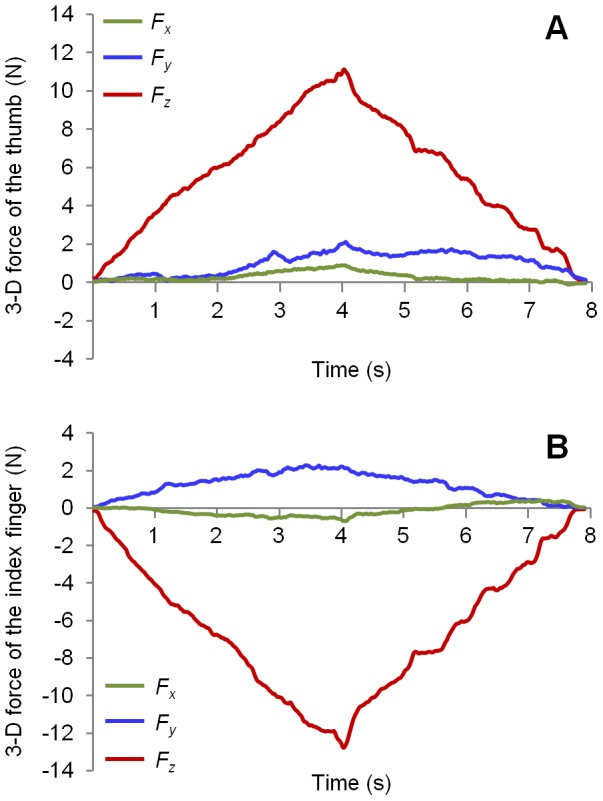
Force vector components of the thumb and index finger from one representative trial. (A) The *F_x_*, *F_y_* and *F_z_* components of the thumb force vector; (B) The *F_x_*, *F_y_* and *F_z_* components of the index finger force vector.

The force vector components (*F_x_*, *F_y_*, and *F_z_*) of the thumb and the index finger expressed in the common coordinate system from one representative trial are depicted in **Figure 3A** and **Figure3B**, respectively. In general, the two digits applied equal normal force magnitudes while following the ramp profile. The *F_y_* shear force components applied by the two digits were in the same direction, whereas the *F_x_* shear force components were in opposite directions and had a lower magnitude than that of the *F_y_* components. The normal forces (F_z_) applied by the thumb and the index finger were opposite in direction and approximately equal in magnitude.

**Figure 4:**
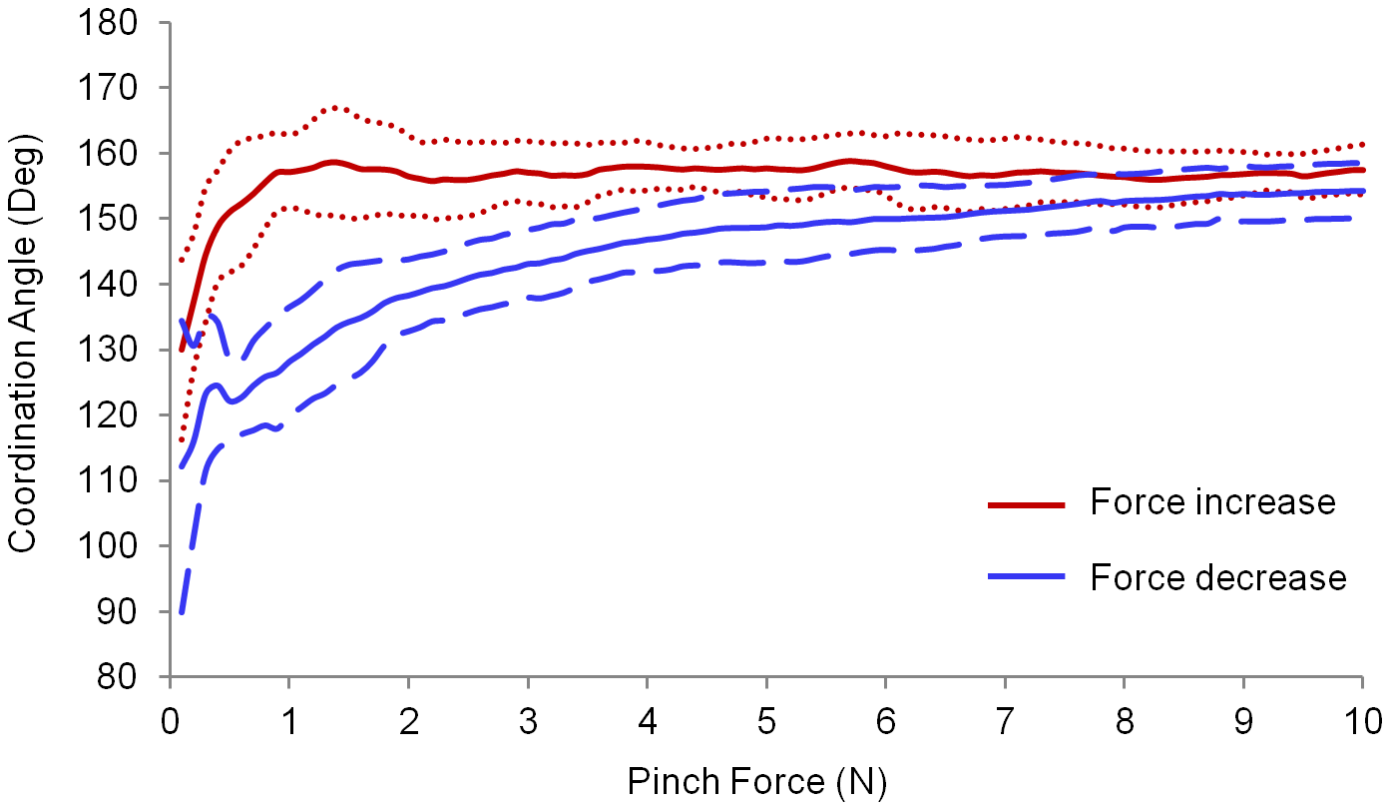
Coordination angles of the two force vectors at different pinch force magnitudes during increasing and decreasing force conditions. The solid lines represent the average of CA across all trials. The dotted/dashed lines represent the plus/minus standard deviations of CA for all trials.

The CA profiles for all subjects during increasing and decreasing pinch force conditions are shown in **Figure 4**. Two characteristics were readily evident. Firstly, the CA nonlinearly changed with pinch force magnitude. When the pinch force increased from 1 to 2 N, the CA rapidly rose from 148.8±8.9° (mean ± standard deviation) to 157.7±7.0°, and when the force increased from 2 N to 10 N, the CA stabilized at about 157°. By contrast, the CA decreased from 154.4±3.8° to 134.4±7.6° with decreasing pinch force from 10 to 2 N; the CA rapidly dropped to 122.2±10.1° when force magnitude was at 1 N. Secondly, a *hysteresis* profile was exhibited in the force-CA relationship across the increasing and decreasing force ramps. The fitted regression model equation of the CA as a function of increasing pinch force was 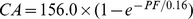 *(R^2^* = 0.908*)* and that of decreasing pinch force was
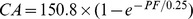
(*R^2^* = 0.816). The two-way repeated measures ANOVA showed main effects of the force level (*F*_2,261_ = 261.8, *p*<0.001) and of the force condition (*F*_1,139_ = 96.9, *p*<0.001), as well as a significant force level × force ramp interaction (*F*_2, 229_ = 19.2, *p*<0.001) on the CA. The upper limit of the CA (parameter A) with the increasing force ramp was 156.0±6.6°, significantly higher than 150.8±9.3° of the decreasing force ramp (*t* = 8.5, *p*<0.001). The fitted force constant value (parameter B) was 0.16±0.13 N for the increasing force ramp, which was significantly lower than the value of 0.25±0.27 N for the decreasing force ramp (*t* = 4.8, *p*<0.001).

**Figure 5:**
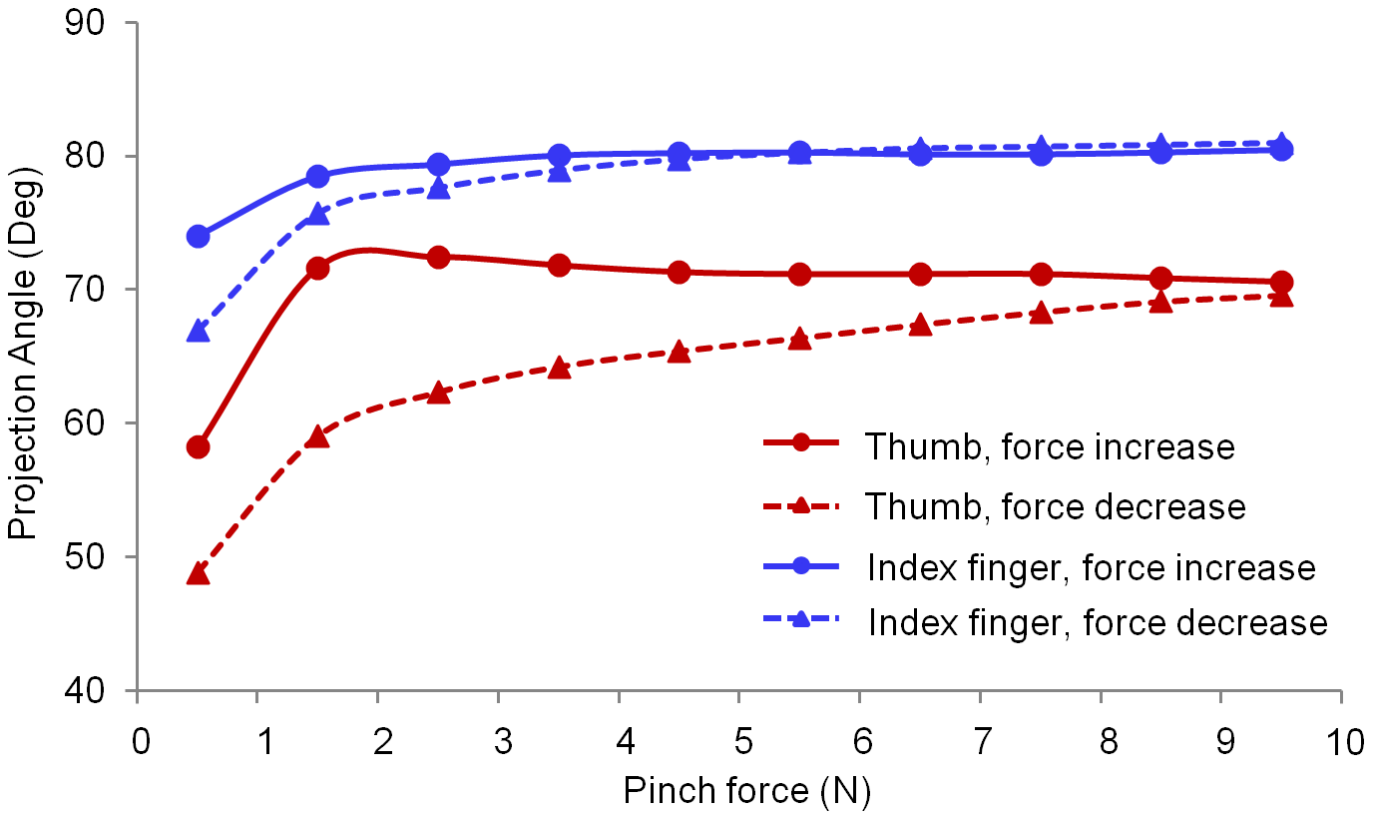
Force projection angles for the thumb and index finger. Each data point represents the average of PA for all trials within the specified 1-N force interval.

The mean absolute values of the PA for all the subjects are shown in **Figure 5**.The three-way repeated measures ANOVA showed significant effects across digit (*F*_1,139_ = 341.7, *p*<0.001), force ramp (*F*_1,139_ = 80.3, *p*<0.001), and force level (*F*_2,233_ = 269.5, *p*<0.001) for the magnitude of the PA. A significant interaction was observed between the digit, the force ramp, and the force level (*F*_2, 261_ = 14.2, *p*<0.001). The absolute PA values for the index finger were higher than those for the thumb, with a mean difference 11.7°. Also, the PA exhibited higher absolute values with the increasing force ramp compared to the decreasing force ramp with a mean difference of 3.5°. For both digits, pairwise comparisons revealed that the PA between 1and 2 N was significantly higher than that between 0 and 1 N for the increasing pinch force condition (*p*<0.001). No significant differences were observed in the PA when the pinch force exceeded 2 N. By contrast, the PA significantly decreased at each force level throughout the decreasing pinch force ramp (*p*<0.001).

**Figure 6:**
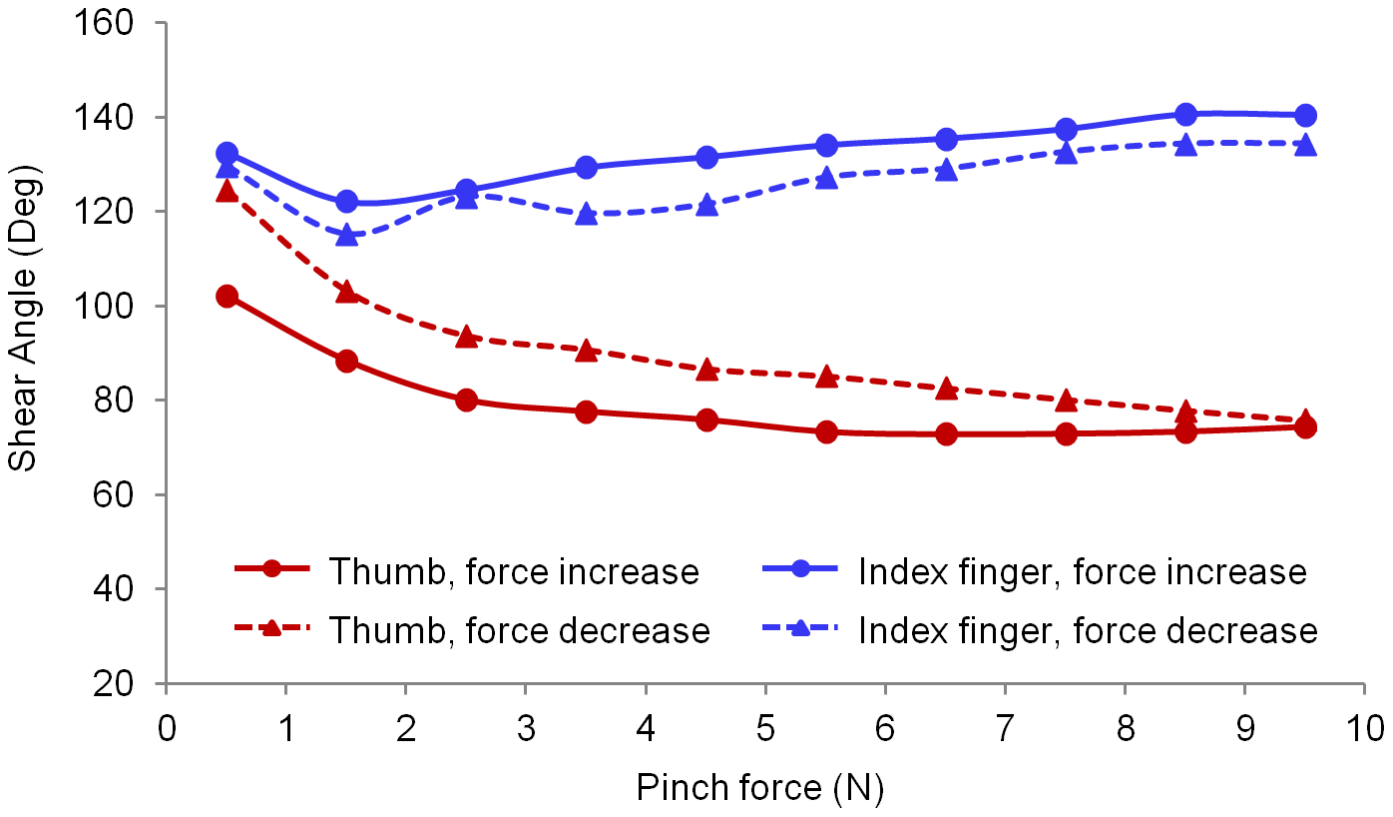
Projection angle for the thumb and index finger. Each data point represents the average of SA for all the trials within the specified 1-N force interval.

**Figure 7:**
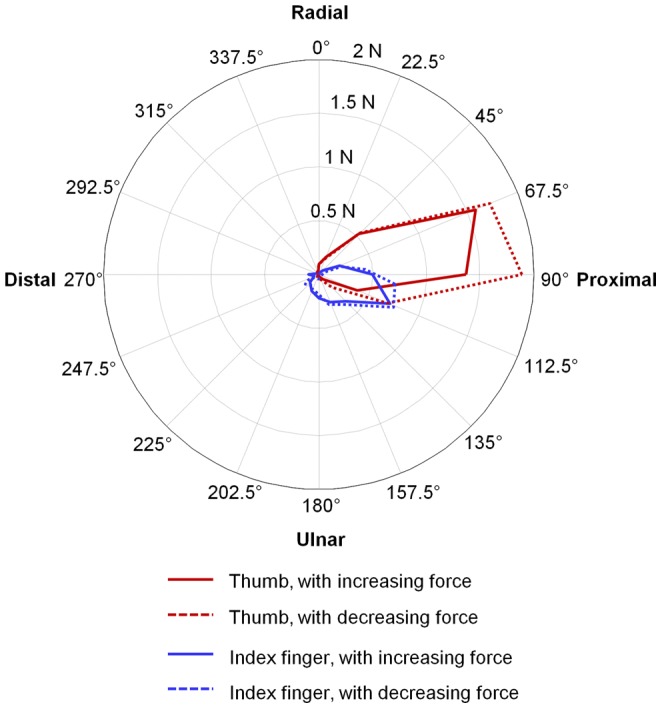
Shear force tuning curves for the thumb and index finger.

The average SA values of all the subjects during the pinch task are shown in **Figure 6**. The three-way repeated measures ANOVA showed significant effects across digit (*F*_1,139_ = 90.6, *p*<0.001) and force level (*F*_3,398_ = 20.0, *p*<0.001) for the SA. The mean SA values of thumb were between 72.9° and 124.6°, whereas that of the index finger ranged from 119.6° to 140.6°. No significant difference of the SA was observed between the increasing and decreasing pinch force conditions (*p* = 0.278). There were significant interactions between the digit and the force ramp (*F*_1,139_ = 15.9, *p*<0.001), and between the digit and the force level (*F*_3, 414_ = 44.7, *p*<0.001) for the SA. The tuning curve shows the distribution of the shear forces on the common shear plane (**Figure 7**). For both increasing and decreasing force conditions, the highest shear force intensity of the thumb was observed to be between 67.5° and 90°, whereas that of the index finger was between 90° and 112.5°. The shear force distribution of the thumb was larger than that of the index finger. Compared to the increasing force ramp, the decreasing force ramp demonstrated larger distribution areas for both digits (**Figure 7**).

## Discussion

This study investigated the force directions applied by the thumb and index finger during precision pinch on an externally stabilized object. The force coordination between the two digits was quantified by the angle formed by their force vectors. Orientations of individual force vectors were analyzed by PAs with respect to the shear plane and SAs in the shear plane. The relationship between the coordination angle and pinch force magnitude was characterized by an exponential function.

### Dependence of force direction on force magnitude

The angular coordination between the force vectors and the orientation of individual force vectors were dependent on the magnitude of pinch force applied, especially at force levels below 2 N. For example during the increasing force ramp condition, the CA was 148.8±8.9° when the pinch force was 1 N, and the angle changed to 157.7±7.0° when the pinch force was 2 N. This force-dependent variation of the CA means that the individual force vectors did not maintain a constant direction or that they did not co-vary in a fixed relationship. In fact, the force vector of the thumb had a greater change of direction than that of the index finger. This is demonstrated by the PA of the thumb force vector which was 62.4±16.7° at 1 N and increased to 72.1±12.0° at 2 N, a change of 9.7° for an increase of 1 N. In comparison, the PA for the index finger force vector changed to a lesser extent (2.9°) when the force increased from 1 N to 2 N. The changes in PA for the thumb and the index finger are indicative of re-orientation of their force vector closer to the direction perpendicular to the shear plane with increasing pinch force magnitude. The change of force vector orientation may be related to the responses of the mechanoreceptors and afferents arising from the tissue deformation associated with the amount of pinch force applied by the digit pulps. It was biomechanically shown that 82% and 92% of fingertip pulp displacement occurred when the grip force magnitude reached to 1 N and 2 N, respectively [12]. Additionally, the responses of the mechanoreceptors induced by the pulp displacement reach marked saturation at 1–2 N grip force [13]. Most daily manipulations are associated with low force applications between 1 and 4 N [14]; therefore, the sensitive responses of the mechanoreceptors to low force application is advantageous for successful performance of those dexterous tasks. In the current study, although the object was fixed, the shear force components associated with the force exertion may have stimulated the directionally-sensitive tactile afferents [15]. Therefore, the mechanoreceptors in the digit pulps likely played a critical role in sensing force application and facilitated digit force coordination with more aligned force vectors when the thumb and the index finger opposed each other during precision pinch.

In addition to the PAs, the force vector directions in the shear plane were examined using the SAs. For both digits, the SA values were in the range of 72.9° to 140.6°, indicating that the shear forces biased towards the proximal direction and acted in an additive rather than canceling manner in this direction. In the other shear direction, the SA showed that the thumb changed its force from ulnar-proximal direction (SA>90°) to radial-proximal direction (SA<90°). By contrast, the index finger consistently oriented its force in the ulnar-proximal direction, which agrees to the observation that during a pressing task with the index finger the digit force vector deviated in the proximal and ulnar direction [10]. The tuning curve showed that the thumb exerted greater shear forces than the index finger, which may be explained by the digits' differences in positional and musculature configurations. For a precision pinch task, the thumb is more obliquely oriented with respect to the contact planes than the index finger. The thumb is equipped with stronger musculature than the index finger, and the collective relative tension capacity of all thumb muscles is 15.2% while the muscles for the index finger have a total tension capacity of 10.4% [16]. The larger shear force at the thumb may also be attributed to the different intrinsic-extrinsic muscle proportions between the two digits. It has been found that intrinsic muscles are highly relevant to digit force direction whereas extrinsic muscles play a key role to stabilize the joints [17].Compared to the index finger, the thumb has more intrinsic muscles to provide greater grasping strength and directional dexterity for precision pinch.

Although the two digits reoriented their force directions with increasing pinch force, the two force vectors did not reach to a perfect opposition alignment of 180°. The perfect alignment of digit force vectors in functional primitives of pinch provided a reference to the force orientation and coordination, which means only normal forces were to be applied as required by the experimental task. The difference between the actual CA and the optimal force orientation of 180° provides quantitative assessment of hand function. For example, clinical studies have found that the extent to which the digit force vectors deviated from the perpendicular direction to the contact surface correlates to the severity of motor deficits associated with stroke [9] or ageing [10]. The asymptotic value (parameter A) derived by the exponential regression model for the force increasing condition was 156.0°, a deviation of 24.0° from perfect alignment. The asymptotic value was even lower (150.8°) for the descending force condition. Likewise, the individual digits did not apply forces perpendicular to the shear plane, although the deviations from the perpendicular direction decreased with increasing pinch forces. The thumb and the index finger approached PA values of 71.1° and 80.5°, which were 18.9° and 9.5° from the perpendicular direction (i.e. 90°).

### Regulation of digit forces

This digit force deviation is due to the muscle activation patterns as the contraction of different muscles can produce fingertip force vectors in different directions [17]–[19]. With abnormal muscle activity patterns, this deflection of fingertip force can be exacerbated. For example, Seo et al. [9] found that when stroke survivors performed a pinch, their extensor digitorumcommunis (EDC) and first dorsalintersseous (FDI) muscles were less activated,but their flexor digitorumsuperficialis (FDS) muscle was overactivated, which led to 2.5 times greater fingertip force deviation than healthy subjects. Additionally, the tactile sensation would also contribute to the fingertip force deflection. It has been shown that the responses of afferents are sensitive to the directions of the tangential force components applied onto the fingertip [15]. Digits need a certain amount of tangential force to resolve the direction of the force vector applied on the fingertip [15], [20]. Reduced tactile sensitivity may hinder the adjustment of fingertip force towards the appropriate direction. For example, with age-related degradation of tactile sensation, the elderly subjects more greatly inclined their force vector, in comparison to younger subjects, when pressing against a force plate with their index finger [10].

### Hysteresis effect of pinch force control

The angular coordination between the two force vectors is not only dependent on the pinching force magnitude, but also influenced by the force history (i.e. ascending from lower force or descending from higher force). During the force increase and decrease cycle, the coordination angle in the decreasing phase showed a higher changing rate with respect to the force magnitudes than in the increasing phase. For example, the CA reached 63% of the plateau values at pinch forces of 0.16 N and 0.25 N (i.e. B of the exponential function) for the force increasing and decreasing conditions, respectively. This suggests that the force vector directions of the thumb and the index finger are less stably controlled in force relaxation than force exertion. Previous studies also shown that decreasing the force magnitude is less involved in online feedback control, and has worse accuracy in force production in comparison to increasing force magnitude profiles [21], [22]. This behavioral hysteresis phenomenon is functionally relevant because increases in force magnitude are more associated with grasping or manipulating objects that require force control for demanding task needs, while decreases in force magnitude are associated with dropping or releasing objects which require a less critical need of force control [23], [24].

### Effects of mechanical constraints on pinch force control

Thumb-finger opposition for precision pinch is considered a hallmark of dexterous tasks completed by the human hand [25]. The thumb pronates and flexes while the index finger flexes to assume an opposition posture for subsequent manipulation. Precision pinch has been examined in many studies with pinch and lift tasks that emphasize coordination between the pinch force and object load [6], [24]. In contrast, our study investigated the force coordination of the thumb and index finger when the pinched object is externally stabilized, which provided an opportunity to observe how the digits naturally coordinate their force vectors without satisfying the requirement of static/dynamic equilibrium [7]. Conceivably, the thumb and the index finger are capable of orienting their force vectors in opposite alignment to balance an object with rotational degrees of freedom. However, the directional coordination between the force vectors was far from alignment (>30° deviation) when object balance is not a performance requirement.

Digit forces during a precision pinch are likely governed by digit-specific force control, inter-digit coordination, and task-dependent force adaptation. First, the thumb and index finger are distinctly different in their anatomical composition, biomechanical function and neuromuscular control. It is expected that each digit has its own preferred force vector output at the digit tip [3], [26]–[29]. This was shown by the greatest misalignment of force vectors at the beginning phase of the pinch when the pinch force was minimal and the CA was low. Second, human digits have built-in anatomical and neural connections that pre-determine synergistic modes for force coordination. For example, it was shown that during a simple pressing task the four fingers shared the total force at the same respective percentages regardless of force magnitude [30]. It was suggested that such a force synergy was to minimize the secondary pronation/supination moment with respect to the longitudinal, neutral axis [30]. In contrast to four-finger pressing, precision pinch is achieved by the two digits opposing each other. When pinching on an object that is externally fixed, the thumb and the index finger chose to stabilize their force directions that are not necessarily preferred for each digit alone, but indicative of thumb-finger coordination. Third, human digits are equipped with independent control and flexible synergies [31], allowing digits to adjust forces for the satisfaction of task constraints [3], [32]. While the extrinsic factor of task constraints can be infinite, the digits have preferred and flexible control to adapt to task requirements.

In summary, this study examined the directions of the force vectors applied by the thumb and the index finger as a function of force production during precision pinch of an externally stabilized object. The directions of force vectors were dependent on the magnitude of the pinch force applied, especially at force levels below 2 N. The two force vectors were never observed to be perfectly opposed (i.e., alignment of 180°), whereby not intrinsically satisfying static equilibrium. In the shear plane, the index finger force vector deviated in the ulnar-proximal direction, whereas the thumb switched its force between the ulnar-proximal and radial-proximal directions. The force vector directions between the thumb and the index finger were uniquely coordinated for force exertion and force relaxation. The digit forces during precision pinch are governed by digit-specific force control, inter-digit coordination, and task-dependent force adaptation. The anatomical composition, biomechanical function, neuromuscular control, and task constraints may collectively influence the directions of digit force vectors during precision pinch. The current findings improve the understanding of digit force control for dexterous manipulation.
